# Effects of different intensities of long-term grazing on plant diversity, biomass and carbon stock in alpine shrubland on the Qinghai-Tibetan Plateau

**DOI:** 10.7717/peerj.12771

**Published:** 2022-01-12

**Authors:** Jinlan Wang, Wen Li, Wenxia Cao, Shilin Wang

**Affiliations:** 1 Gansu Agricultural University, Grassland Science College, Lanzhou, Gansu, China; 2 Qinghai University, Qinghai Academy of Animal Science and Veterinary Medicine, Xining, Qinghai, China

**Keywords:** Grazing intensity, Carbon storage, Alpine shrubland, Qinghai-Tibetan Plateau

## Abstract

Grazing is the main grassland management strategy applied in alpine shrubland ecosystems on the Qinghai-Tibetan Plateau. However, how different intensities of long-term grazing affect plant diversity, biomass accumulation and carbon (C) stock in these ecosystems is poorly understood. In this study, alpine shrubland with different long-term (more than 30 years) grazing intensities (excluded from grazing for 5 years (EX), light grazing (LG), moderate grazing (MG) and heavy grazing (HG)) on the Qinghai-Tibetan Plateau were selected to study changes in plant diversity, aboveground biomass and C accumulation, as well as distribution of C stock among biomass components and soil depths. A structural equation model was used to illustrate the impact of grazing on the soil carbon stock (SOC). The results showed that the Shannon–Wiener diversity index and richness index of herbaceous plants, shrubs, and communities first significantly increased and then decreased with increasing grazing intensity, reaching maxima at the LG site. The aboveground and belowground and litter biomass of understory herbaceous plants, shrubs and communities decreased with increasing grazing intensity, reaching maxima at the EX site. The aboveground and belowground biomass C storage decreased with increasing grazing intensity, reaching maxima at the EX site. The SOC stock and total ecosystem C stock decreased with increasing grazing intensity, reaching maxima at the EX and LG sites. A structural equation model showed that grazing-induced changes in the belowground biomass of understory herbaceous plants greatly contributed to the SOC stock decrease. Thus, considering the utilization and renewal of grassland resources, as well as local economic benefits and ecological effects, LG may be a more rational grazing intensity for species diversity conservation and ecosystem C sequestration in alpine shrubland. Our results provide new insights for incorporating grazing intensity into shrub ecosystem C stock and optimizing grazing management and grassland ecosystem C management.

## Introduction

Grasslands, which constitute the second largest global ecosystem, not only provide economic and recreational value but also perform critical ecosystem services, such as carbon (C) sequestration and mitigating global climate change (David et al., 2018; Fan et al., 2019). Across the globe, grassland ecosystems store approximately 34% of the global soil organic carbon (SOC) and thus play an important role in the global C cycle (Lorenz & Lal, 2018; Viglizzo et al., 2019). In recent years, grassland degradation has been exacerbated by human disturbance (*e.g.*, overgrazing) and rapid climate change, which causing considerable losses of SOC (Liu, Yang & Zhang, 2015; Georg et al., 2019; Tian et al., 2020). Even a small change in grassland SOC stock could significantly alter the concentration of CO_2_ in the atmosphere and help mitigate global climate change (Zhao et al., 2017; Wang et al., 2019). Nevertheless, SOC is a potentially manageable resource, and the loss of C from soil could be regained by improved management (Six et al., 2007). Therefore, it is crucial to understand how SOC storage responds to grassland management practices to better predict the regional C balance and grassland sustainable management (Li et al., 2018a; Yu et al., 2020).

Grazing is one of the most important land management practices in grasslands worldwide (Zhou et al., 2017). Livestock grazing influences grassland SOC dynamics mainly through impacts on plant C allocation patterns and soil characteristic availability due to soil compaction by livestock trampling (Wilson et al., 2018). However, the impacts of grazing on grassland SOC storage are controversial (Sun, Ma & Lu, 2018). For example, grazing has various effects on the SOC stock in grassland ecosystems, with positive (Hewins et al., 2018; Dong et al., 2020; Wei et al., 2021), neutral (Fan et al., 2021) and negative effects (Liu et al., 2021). Nevertheless, the impact of grazing on grassland SOC is a very complex process regulated by a range of environmental factors (*e.g.*, grassland ecotype, precipitation and temperature) and management practices (*e.g.*, grazing intensity and duration (Jiang et al., 2020). In addition, grazing has a hysteretic effect on grassland soil systems, and only on a long time scale can the ecological effects of grazing be fully reflected (Yang et al., 2013). Despite a great deal of literature about how to best manage livestock grazing to achieve ecological and/or production functions (Li et al., 2017; Sun, Ma & Lu, 2018; Cdab et al., 2020), how grazing, especially its intensity, influences the distribution and sequestration of C over a longer time scale remains considerably unclear (Zhou et al., 2017; Zubieta et al., 2021). Therefore, the development of sustainable grazing management strategies over long time scales, especially the optimal grazing intensity, that increases vegetation diversity and productivity, enhance ecosystem function and services is becoming a global concern (Pakeman et al., 2019).

Alpine shrubland is a widely distributed grassland type on the Qinghai-Tibet Plateau, and accumulated large amounts of SOC due to high altitude and lower temperatures (Zhao et al., 2010; Nie et al., 2019). The shrubland is considered the largest uncertain factor in the C balance in terrestrial ecosystems (Piao et al., 2010). Moreover, the alpine shrubland on the Qinghai-Tibet Plateau has been severely degraded due to overgrazing and rapid global climate change, which greatly decreases plant diversity, productivity, ecosystem services and sustainability (Mhsa et al., 2020). Furthermore, alpine shrubland is more vulnerable to climate change and human disturbance than other alpine grassland ecosystems on the Qinghai-Tibet Plateau (Yang et al., 2009). Therefore, it is essential to explore the appropriate grazing management practices, especially the optimal grazing intensity for alpine shrubland ecosystems against the background of global warming, to better understand the role of shrubland ecosystems in the terrestrial ecosystem C balance. Currently, there are few studies on the effect of long-term grazing on alpine shrubland, and little is known about the dynamic changes in the SOC pool in alpine shrubland under different grazing intensities over a long time scale; thus, further research is needed. Therefore, precise quantification of the C dynamics of alpine shrubland ecosystems under different grazing intensities is required to better predict the regional C balance and sustainable grassland management over a long time scale.

In the current study, we compared the plant biomass C and soil C stocks under different long-term (more than 30 years) grazing intensities (excluded from grazing for 5 years (EX), light grazing (LG), medium grazing (MG) and heavy grazing (HG)) in an alpine shrubland on the Qinghai-Tibet Plateau. We hypothesized that the plant diversity, biomass and C storage will exhibit a first significantly increased and then decreased pattern with increasing grazing intensity. The focus of our research was to explore how different grazing intensities affect C storage in alpine shrubland ecosystems to predict the C balance and to optimize grazing management strategies to minimize the impacts of climate change on alpine shrubland to ensure their sustainable development.

## Materials and methods occurs

### Study area

**Figure 1:**
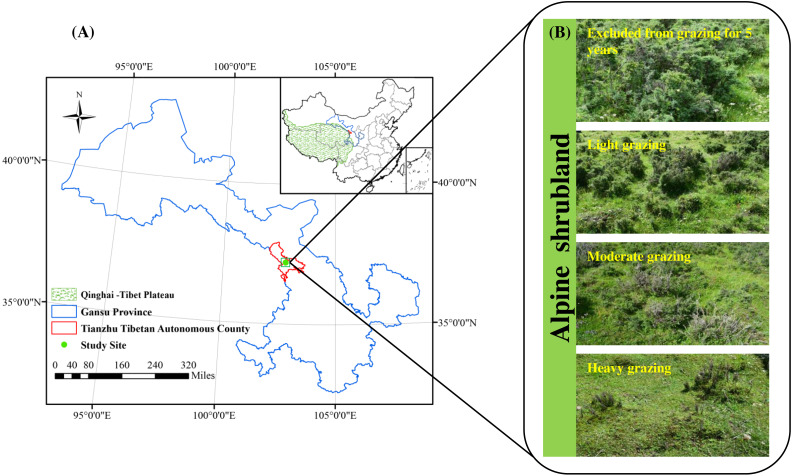
Location of the study area (A) and study treatments (B).

Research was conducted in Qilian Mountain National Park located in Tianzhu Tibetan Autonomous County (N37°10′16.97″, E102°47′17.31″, 3,050 m of altitude), Gansu Province, China (Fig. 1A). This area has a plateau-continental climate with a high altitude (above 3200 m), low air oxygen content and temperature, intense ultraviolet radiation and short plant growing period (120 days). The average annual air temperature is 0.16 °C, ranging from a maximum of 11.2 °C in July to a minimum of −11.4 °C in January. The mean annual rainfall is 415.0 mm, of which 76% falls during June–September (Wang et al., 2020). The soil is an alpine chernozem soil. The vegetation type is alpine shrubland, and the dominant species are *Rhododendron thymifolium*, *Rhododendron capitatum* and *Salix oritrepha*, and the companion species are *Potentilla fruticosa*, *Caragana jubata*, *Rhododendron anthopogonoides* and *Spiraea alpina*. The grassland in the study area belongs to lightly degraded according to the alpine grassland degradation criteria, and serves as the local winter and spring pasture (grazing rest during the other time). The proportion of shrubs and herbs is approximately 40% and 80%, respectively. In September 2019, when the alpine vegetation had reached its maximum biomass, we calculated that the theoretical carrying capacity of the alpine shrubland in the study area was 3.7 AUM. ha^−1^ based on the 50% utilization rate. An AUM is the amount of forage required by a 454 kg cow (requires about 11.8 kg of dry matter forage per day) for one month (Jacoby, 1989).

### Experimental design

The alpine shrubland in our research area was a public grassland before 1984, and was managed by individual households of herdsmen due to the implementation of the household responsibility system in 1984. The fences were built since 1984. Therefore, a natural grazing intensity gradient was formed due to the changes in grazing time and livestock number. We chose three shady slopes (25–34°) as three study sites in September 2019, and all sites were located at least 1.0 km away from each other. The soil type, vegetation community composition and topography are basically the same among the three sites. Our study design consisted of four grazing intensities, including excluded from grazing for 5 years (EX), lightly grazed (LG), moderately grazed (MG) and heavily grazed (HG) alpine shrubland (Fig. 1B). The four grazed treatments were randomly assigned within each study site in a randomized complete block design. The area of each grazed plot was approximately 0.6–0.8 ha. All plots were located at least 60 m between any two plots. From 1984 to 2019, the LG treatment was grazed freely by Tibetan sheep and yaks as spring-autumn pastures at intensities of 2.6 ± 0.23 AUM. ha^−1^, which was below the theoretical capacity. The MG treatment was grazed freely by Tibetan sheep and yaks as spring-autumn pastures at intensities of 3.9 ± 0.29 AUM. ha^−1^, which was basically close to the theoretical capacity. The HG site continued grazing by Tibetan sheep and yaks throughout the year from 1984 to 2019 at intensities of 7.6 ± 0.37 AUM. ha^−1^ (approximately two times the theoretical capacity). The EX site was absolutely excluded from livestock grazing all year round for more than 5 years. The vegetation and soil characteristics of the study sites are shown in Table 1.

**Table 1:** Description of the basic situation of different sampling plots.

		Grazing intensity			
			EX	LG	MG	HG
Shrub		Coverage (%)	92.3 ± 0.87a	88.9 ± 0.92b	82.0 ± 0.97c	7.7 ± 0.65d
		Height (cm)	78.6 ± 1.16a	65 ± 2.04b	51.5 ± 1.28c	11.7 ± 1.05d
		Density (individual/m^2^)	3.4 ± 0.14a	3.1 ± 0.11a	2.66 ± 0.19b	1.18 ± 0.11c
		Dominant species	*Rhododendron capitatum*, *Salix oritrepha*	*Rhododendron capitatum*, *Salix oritrepha*	*Rhododendron capitatum*, *Rhododendron thymifolium*	*Potentilla* *fruticosa*
Understory herbs		Coverage (%)	90.8 ± 1.0a	85.5 ± 1.6b	86.6 ± 2.1ab	61.0 ± 1.9c
		Height (cm)	32.8 ± 1.3a	21.4 ± 0.8b	8.6 ± 0.3c	1.8 ± 0.1d
		Density (individual/m^2^)	614.8 ± 27.4b	711.4 ± 27.2a	657.7 ± 17.4ab	399.5 ± 40.6c
		Dominant species	*Polygonum viviparum*	*Elymus nutans*	*Elymus nutans*	*Kobresia humilis*
Soil	0–10 cm	Bulk density (g/cm^3^)	0.8 ± 0.01c	0.8 ± 0.01c	0.9 ± 0.02b	1.2 ± 0.04a
		Soil organic carbon (g/kg)	110.8 ± 3.8a	111.6 ± 2.2a	85.8 ± 1.9b	39.5 ± 1.5c
	10–20 cm	Bulk density (g/cm^3^)	0.8 ± 0.01b	0.8 ± 0.01b	0.9 ± 0.04b	1.1 ± 0.1a
		Soil organic carbon (g/kg)	89.5 ± 1.9a	89.6 ± 1.3a	73.7 ± 2.1b	39.3 ± 8.4c
	20–30 cm	Bulk density (g/cm^3^)	0.9 ± 0.01b	0.8 ± 0.01c	0.9 ± 0.03bc	1.1 ± 0.03a
		Soil organic carbon (g/kg)	55.0 ± 2.2a	55.9 ± 0.8a	48.3 ± 2.3b	30.6 ± 0.7c
	30–50 cm	Bulk density (g/cm^3^)	1.21 ± 0.04a	1.2 ± 0.1a	1.3 ± 0.1a	1.2 ± 0.1a
		Soil organic carbon (g/kg)	35.5 ± 1.8a	34.3 ± 2.3a	35.1 ± 0.1a	28.1 ± 0.8b

**Notes.**

EX excluded from grazing for 5 years LG lightly grazed MG moderately grazed HG heavily grazed BD soil bulk density SM soil moisture SOC soil organic carbon TN soil total nitrogen

Different lowercase letters in the same line indicate significant differences at different grazing intensities *P* < 0.05.

### Field measurements and sampling

During the period from early September to middle September in 2019, when the alpine shrubs reached their peak biomass (Wang et al., 2021), ten quadrats (5 m ×5 m) were randomly established approximately 1.5 m from the edge in each grazed plot to investigate shrub community structure. We surveyed 40 quadrats per grazed treatment and 120 quadrats in total. The height, coverage and number (density) of each shrub were measured in each quadrat in the field. In addition, we chose one standard shrub for each shrub species in each quadrat and then completely excavated them. According to the method of Deng et al. (2017), we divided the standard shrubs into four components: leaf, branch, stem and root. We weighed the fresh weight of all above- and belowground components immediately by using portable scales *in situ* and then collected 300–500 g samples of each component in every standard shrub for moisture and organic carbon content measurement. In addition, we randomly established ten 50 cm × 50 cm small quadrats (1.5 m from the edge) in each grazed plot to investigate the aboveground biomass and litter biomass of the understory herbs community (40 quadrats per treatment, and 120 quadrats in total). All aboveground green herbs were cut at ground level, and the litter was collected by hand in each small quadrat.

We collected soil samples in the 0–10, 10–20, 20–30 and 30–50 cm soil layers by using a 10-cm inner diameter auger in each small quadrat to measure the root biomass of the herbs. Two cores were collected from each small quadrat and then mixed together to form one composite sample (10 composite samples per grazed plot and 40 composite samples in each treatment). We separated the roots by washing the soil samples within a 0.5 mm mesh bag. The shrub and herbs above- and belowground samples were immediately dried at 105 °C for 30 min and then oven-dried at 70 °C until constant weight. The soil bulk density of the samples from the 0–10, 10–20, 20–30 and 30–50 cm soil layers was measured by using a cutting ring (100 cm^3^ volume) in each of the harvested quadrats (10 per grazed plot, and 30 per treatment).

### Diversity calculation

The Shannon–Wiener diversity index (the diversity of species within a community or habitat, *H*), evenness index (distribution of the number of individuals per species in a community or habitat, *J*), and richness index (the number of species in a community or habitat, *S*) of understory herbs and shrubs were calculated using the methods of Lindsey (1956).

### Organic carbon content analysis

The above- and belowground parts of herbs and different shrub components were crushed and sieved through a 0.5 mm mesh sieve for testing organic carbon after determining the dry weight. The organic carbon contents of the soil and vegetation were assayed by using the dichromate oxidation method (Bao, 2000).

### Organic carbon storage calculation

The vegetation biomass organic carbon storage was calculated with the following equation (Deng et al., 2017):

(1) \begin{eqnarray*}{\mathrm{CS}}_{\mathrm{V }}={\mathrm{B}}_{\mathrm{V }}\times {\mathrm{C}}_{\mathrm{V }}\end{eqnarray*}

where CS_V_ is vegetation biomass organic carbon storage (g/m^2^), BV is vegetation biomass (g/m^2^) and C_V_ is the vegetation organic carbon concentration (%).

The soil organic carbon storage was calculated with the following equation (Deng et al., 2017):

(2) \begin{eqnarray*}{\mathrm{CS}}_{\mathrm{S}}=\mathrm{BD}\times {\mathrm{SOC}}_{\mathrm{C}}\times \mathrm{ST}\times 10\end{eqnarray*}

where CS_S_ is soil organic carbon storage (g/m^2^), BD is soil bulk density (g/m^3^), SOC_C_ is soil organic carbon content (g/kg) and ST is soil thickness (cm).

Ecosystem organic carbon storage was considered the sum of the plant, litter, root and SOC storage.

### Statistical analysis

Using the following polynomial regression, we explored the relationships between grazing intensity and understory herbs, shrubs, and community Shannon–Wiener diversity index, evenness index and richness index:

(3) \begin{eqnarray*}y=a{x}^{2}+bx+c\end{eqnarray*}

where y is the Shannon–Wiener diversity index, evenness index or richness index, x is grazing intensity, and a, b and c are fitted coefficients.

Using the following polynomial regression, we explored the relationships between grazing intensity and biomass C storage, SOC storage and ecosystem C storage:

(4) \begin{eqnarray*}y=d{x}^{2}+ex+f\end{eqnarray*}

where y is the biomass C storage, SOC storage or ecosystem C storage, x is grazing intensity, and d, e and f are fitted coefficients.

Kolmogorov–Smirnov and homogeneity of variance analyses were used to test the normal distribution and homogeneity of variance of diversity, biomass, organic carbon content and organic carbon storage, respectively. Then, we used one-way analyses of variance followed by Tukey’s multiple comparisons test to compare the significant differences in diversity, biomass, organic carbon content and organic carbon storage among different grazing treatments at a significance level of *P* < 0.05. Structural equation modeling (SEM) was used to test the direct and indirect effects of grazing, soil water content, above- and belowground biomass of understory herbs, understory herbs diversity and litter biomass on SOC storage. All statistical analyses were performed with the SPSS software program ver. 19.0 (SPSS, Chicago, Illinois, USA). All figures were generated with Origin software ver. 8.5, and the SEM model was constructed with R language ver.4.0.2.

## Results

### Diversity and biomass at different grazing intensities

**Figure 2:**
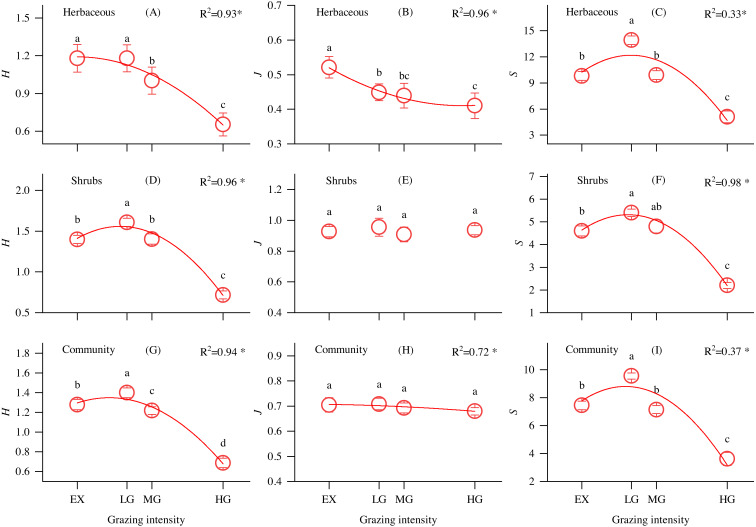
Changes in the understory herbs, shrubs, and community Shannon-Wiener diversity index (*H*), evenness index (*J*) and richness index (*S*) and its relationship with grazing intensity in alpine shrubland under different grazing intensities. Note: EX, excluded from grazing for 5 years; LG, lightly grazed; MG, moderately grazed; HG, heavily grazed. * *P* < 0.05.

As shown in Fig. 2, the Shannon–Wiener diversity index of shrubs (Fig. 2D), communities (Fig. 2G) and richness index of understory herbs (Fig. 2C), shrubs (Fig. 2F), and communities (Fig. 2I) first increased and then decreased with increasing grazing intensity, reaching a maximum at the LG site. The evenness index of understory herbs significantly decreased along the grazing intensity gradient (Fig. 2B), while there were no significant differences in the shrub and community evenness indices at different grazing intensities sites.

The aboveground biomass of understory herbs (UAGB) first increased and then decreased with increasing grazing intensity, reaching a maximum at the LG site of 6503.04 g/m^2^ (Table 2). The belowground biomass of understory herbs (UBGB) and total understory herbs biomass (TUB) significantly decreased with increasing grazing intensity (*P* < 0.05), and the highest values appeared at the EX site (Table 2). In terms of shrubs, there was a significant *decrease* in leaf and branch biomass, belowground biomass of shrubs (SBGB) and total shrub biomass (TSB) with increasing grazing intensity (*P* < 0.05), reaching their maxima at the EX site, and lower values appeared at the HG site (Table 2). The litter biomass (LB) and total biomass (TB) decreased with increasing grazing intensity, with the highest values at the EX site of 523.1 g/m^2^ and 6503.04 g/m^2^, respectively.

**Table 2:** Differences in biomass (g/m^2^) in alpine shrubland with different grazing intensities.

	Grazing intensity			
		EX	LG	MG	HG
Understory herbs	UAGB	108.1 ± 1.5b	122.8 ± 3.5a	70.1 ± 3.8c	78.9 ± 3.7c
	UBGB	1385.4 ± 80.1a	1103.1 ± 29.2b	989.0 ± 43.3b	666.3 ± 15.6c
	TUB	1493.5 ± 81.0a	1225.9 ± 29.9b	1059.1 ± 45.7c	745.2 ± 16.4d
Shrubs	Leaf	260.9 ± 6.5a	226.2 ± 4.2b	160.5 ± 5.6c	10.9 ± 0.7d
	Branch	497.0 ± 10.7a	384.4 ± 6.6b	265.4 ± 6.1c	17.8 ± 1.1d
	Stem	1152.4 ± 27.6a	1196.0 ± 24.1a	959.6 ± 18.4b	73.9 ± 2.8c
	SAGB	1910.3 ± 35.0a	1926.6 ± 28.2a	1385.6 ± 25.8b	102.6 ± 4.0c
	SBGB	2576.1 ± 37.3a	2449.0 ± 48.2b	1798.7 ± 31.7c	212.4 ± 8.6d
	TSB	4486.4 ± 51.0a	4375.6 ± 57.5a	3184.2 ± 38.5b	315.0 ± 10.9c
Litter	LB	523.1 ± 6.1a	167.8 ± 6.3b	86.0 ± 3.3c	10.0 ± 0.6d
Total biomass	TB	6503.0 ± 78.6a	5769.3 ± 74.1b	4329.4 ± 59.9c	1070.2 ± 18.8d

**Notes.**

EX excluded from grazing for 5 years LG lightly grazed MG moderately grazed HG heavily grazed UAGB aboveground biomass of understory herbaceous plants UBGB belowground biomass of understory herbaceous plants TUB total understory herbaceous biomass SAGB aboveground biomass of shrubs SBGB belowground biomass of shrubs TSB total shrub biomass LB litter biomass

Different letters in the same row denote significant differences at *P* < 0.05. The values are mean ± SE.

### Carbon accumulation at different grazing intensities

**Figure 3:**
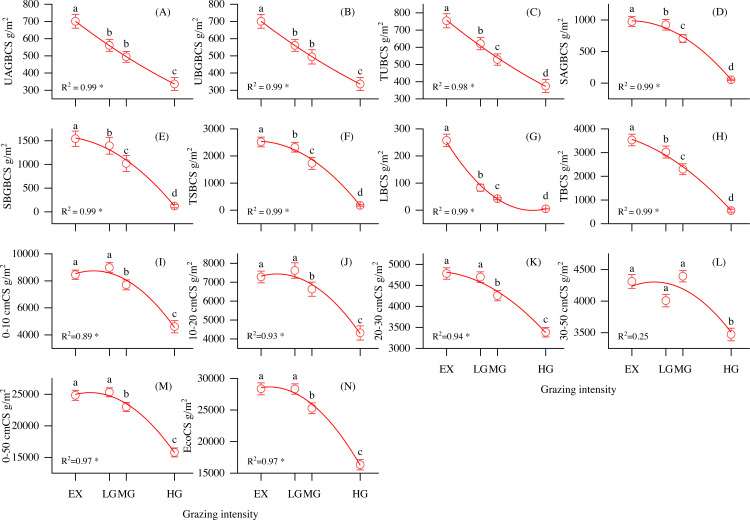
Changes in carbon storage and its relationship with grazing intensity in alpine shrubland under different grazing intensities. Note: UAGBCS, aboveground biomass carbon storage of understory herbaceous plant carbon; UBGBCS, belowground biomass carbon storage of understory herbaceous plants; TUBCS, total understory herbaceous plant biomass carbon storage; SAGBCS, aboveground biomass carbon storage of shrubs; SBGBCS, belowground biomass carbon storage of shrubs; TSBCS, total shrub biomass carbon storage; LBCS, litter biomass carbon storage, TBCS; total vegetation biomass carbon storage; 0–10 cmCS, soil carbon storage in 0–10 cm layer; 10–20 cmCS, soil carbon storage in 10–20 cm layer; 20–30 cmCS, soil carbon storage in 20–30 cm layer; 30–50 cmCS, soil carbon storage in 30–50 cm layer; 0–50 cmCS, soil carbon storage in 0–50 cm layer; EcoCS, ecosystem carbon storage. * *P* < 0.05.

As shown in Fig. 3, the vegetation biomass carbon pool, SOC pool, and ecosystem total carbon pool were strongly correlated with grazing intensity. UAGBCS, UBGBCS, TUBCS, SAGBCS, SBGBCS, TSBCS, LBCS and TBCS decreased with increasing grazing intensity (Figs. 3A–3H). In addition, the 0–10, 10–20, 20–30, 30–50 and 0–50 cm soil layer organic pools decreased with increasing grazing intensity (Figs. 3I–3M). The ecosystem carbon storage significantly decreased with increasing grazing density (*P* < 0.05, *F* = 46), reaching the highest value at the EX site at 28356.07 g/m^2^ (Fig. 3N). Vegetation, root and soil organic carbon accounted for 0.6–4.5%, 2.8–7.9% and 87.5–96.6% of the ecosystem carbon pool, respectively.

### Structural equation model of the effects of grazing on carbon storage

**Figure 4:**
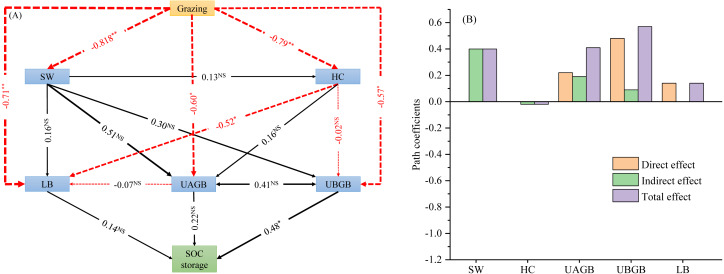
Effects of livestock grazing on soil organic carbon storage calculated by the structural equation model (A). Direct, indirect and total effects of grazing, abiotic and biotic driving factors on soil C storage (B). Note: SW, soil water content; UAGB, aboveground biomass of understory herbaceous plants; UBGB, belowground biomass of understory herbaceous plants; LB, litter biomass; HC, understory herbaceous plant diversity; LB, litter mass. *, *P* < 0.01; **, *P* < 0.001; NS, no significant difference (*P* > 0.05).

The structural equation model for SOC storage showed a good fit (GFI = 0.995, CFI = 0.849, *P* = 0.000, Figs. 4A, 4B). The path analysis of the structural equation model indicated that litter biomass, above- and belowground biomass of understory herbs had impacts on SOC storage (Figs. 4A, 4B). On the basis of the standardized values of statistically significant SEM paths, we obtained the direct, indirect and total effects on SOC storage. Among the effects on SOC storage, UBGB had the highest direct effect and total effect (0.48 and 0.57, respectively). Therefore, Among the total effects on SOC storage, the belowground biomass of understory herbs made a much greater contribution than the soil water content, understory herbaceous diversity, litter biomass, and aboveground biomass of understory herbs (Figs. 4A, 4B).

## Discussion

### Responses of plant diversity and biomass to different grazing intensities

Grazing is a human-related disturbance factor that can substantially affect ecosystem functions, mainly altering vegetation growth and composition by livestock foraging, manure deposition and trampling (Zhou et al., 2017). The grazing gradient, especially on long time scales, is one of the main factors that changes the species diversity of grassland vegetation communities (Freitag et al., 2021). In the current research, the Shannon–Wiener diversity and richness index of understory herbs, shrubs and communities all first significantly increased and then decreased with increasing grazing intensity, reaching their maxima at the LG site. Those results support our hypothesis. Grazing may reduce plant diversity and richness by consuming palatable plant species (Karami et al., 2021). For example, graminoids, which are palatable to herbivores, have been completely removed from grazed sites due to livestock grazing (Li et al., 2018b). In addition, grazing exclusion increased plant coverage and height, especially in dominant species, which might exacerbate the competition for light and other resources among plant species, thus decreasing plant species diversity and richness (Wu et al., 2017). Moreover, litter biomass accumulation may hinder plant reproduction and regeneration, giving rise to community composition change and biodiversity loss (Tian et al., 2020). In the EX site, litter biomass was largely accumulated due to removal of the livestock disturbance, which might have negative impacts on plant diversity and species richness.

Biomass allocation is the crucial index for the determination of community structure and function and for comprehending ecosystem service dynamics (Li et al., 2017; Ji, Li & Li, 2020b). Biomass is mainly determined by local climatic and topographic conditions, soil characteristics and grazing management strategy (Singh et al., 2020). However, biomass in the current research was measured at a small scale, and the differences in biomass were mainly determined by browsing, trampling behavior and manure deposition of livestock grazing. Grazing could reduce the height and coverage of vegetation *via* livestock browsing, finally affecting the total aboveground biomass (Ayuso et al., 2019). In our research, the shrub aboveground biomass, understory herb aboveground biomass and litter biomass at the EX and LG sites were significantly higher than those at the MG and HG sites. Dominant plants at the EX site significantly increased without external interference (herbivore hoof trampling and browsing), thus increasing aboveground biomass and further accumulating litter biomass (Li et al., 2017). In additional, with the increasing of grazing intensity, large areas of photosynthetic organs loss due to grazing, inhibited photosynthesis of these plants, thus reducing the compensation of aboveground biomass, and finally led to the decrease of aboveground biomass (Seabloom, Borer & Tilman, 2020). This phenomenon is most obvious at the HG site, livestock browsing decreased temporary or permanent reproduction and regeneration of plants, leading to lower aboveground and litter biomass (Sun, Ma & Lu, 2018). Below-ground biomass is an important component of grassland ecosystem because of its important process functions, such as regulating plant growth and development, storing nutrients, supplying above-ground water (Laskar et al., 2020). Grazing decreased root biomass compared with nongrazing in the current study, which was more pronounced at the HG site. These results are consistent with those of a previous study (Cdab et al., 2020). This was mainly because trampling by livestock compacted the soil, creating an anaerobic environment that limited plant growth, especially root growth (Sugai et al., 2020).

### Responses of C storage to different grazing intensities

Similar to biomass changes, biomass carbon storage decreased with increasing grazing intensity, and the result does not support our hypothesis. Shrub and understory vegetation fixed carbon mainly through photosynthesis (Griebel et al., 2020). Grazing reduced the biomass of photosynthetic organs, such as leaves, branches and stems, reducing the access rate and the ability of grassland primary productivity to fix carbon (Dibar et al., 2020). Analysis of ecosystem carbon storage revealed that soil was the largest reservoir regardless of the grazing intensity, accounting for 87.5%–96.6% of the ecosystem carbon pool in the current research. In line with previous studies (Zhou et al., 2017; Abdalla et al., 2018), the 0–10, 10–20, 20–30 and 0–50 cm layer SOC storage all showed a decreased pattern with increasing grazing intensity, and the results do not support our hypothesis. In the alpine shrubland, the patterns of SOC stock may be attributed, at least in part, to grazing (Alberti et al., 2017). Livestock grazing, on the one hand, reduced vegetation residues returned to the soil (Ji et al., 2020a), weakened photosynthetic energy to the soil due to greater consumption of photosynthetic tissue (Tian et al., 2020), and enhanced the outflow of energy and nutrition from the soil-grassland ecosystem to primary consumers (Wang, 2016), which reduced potential carbon inputs to the soil. On the other hand, livestock hoof trampling promoted soil respiration and mineralization (Ma, Ding & Li, 2016), thereby increasing soil organic carbon loss, especially at the HG site. Generally, SOC stock are affected by plant aboveground and belowground biomass, litter accumulation and decomposition (Kumar et al., 2021). In the current study, we found that the belowground biomass made a much greater contribution than the litter biomass and aboveground biomass to soil carbon sequestration. This is mainly because of the rapid growth and turnover of fine roots, which have a short life cycle and can decompose into the soil in a short time. In addition, the root mass could directly increase the input of soil organic matter through root exudates. Moreover, the root biomass is much larger than the litter biomass and aboveground biomass (the root/ shoot ratio was ranging from 1.9 to 4.8 in current study), and thus, the greater biomass likely results in more carbon input into the soil.

The alpine shrubland ecosystem carbon pool showed a decreased pattern with increasing grazing intensity, reaching the highest value at the excluded from grazing for 5 years site. The results do not support our hypothesis. Our result was agreed with the result of Han et al. (2008), who reported that the carbon storage declined as grazing intensity increased in the meadow steppe of Inner Mongolia, China. Higher grazing intensity is generally expected to lead to greater organic carbon loss, because greater removal of plant species/photosynthetic tissue and greater reduction of material/energy input, hence reduces C inputs to carbon storage of the ecosystem (Mcsherry & Ritchie, 2013). Grazing exclusion has been widely adopted as a grassland restoration measure in response to the national policy of returning grazing land to grassland in China (Wang et al., 2020). From the perspective of operating cost and ecosystem carbon storage, grazing exclusion within a short period of time (5 years) was a low-cost and most effective approach for grassland restoration. However, some other researchers have believed that ecosystem carbon storage begins to show a downward trend after reaching a certain peak value with the extension of exclusion time (Li et al., 2018b). In addition, considering that a dilemma exists between the utilization and renewal of grassland resources after grazing exclusion, grazing exclusion should be implemented with caution on the Tibetan Plateau and other similar areas (Sun et al., 2020). Although the carbon storage of alpine shrubland ecosystem at the EX site higher than that at LG, MG and HG sites in the current research, LG is a valid grazing management practice for enhancing ecosystem carbon storage in consideration of ecological effects and economic effects. The result has been approved by Li et al. (2021), who suggested that LG was beneficial for the sustainable development of grassland ecosystems.

## Conclusion

A grazing intensity gradient in an alpine shrubland on the Qinghai-Tibetan Plateau resulted in a significant decrease in plant biomass and the plant carbon pool from the EX site to the HG site. Shrubs play a vital role in ecosystem carbon accumulation compared with understory herbs, and the ligneous components (roots, stems and branches) of shrubs significantly decreased with increasing grazing intensity on long time scales. Moreover, SOC accumulation mainly occurred in the surface soil, the SOC stock showed a parabolic decreasing pattern with increasing grazing intensity on long time scales, and the belowground biomass of understory herbs was the driving factor for the SOC stock. In addition, the ecosystem carbon pool showed a parabolic decreasing pattern with increasing grazing intensity on long time scales. The total ecosystem carbon at the EX and LG sites was significantly higher than that at the MG and HG sites, accompanied by the aboveground and belowground biomass and SOC stock. Considering that grazing is the main grassland use in the utilization and renewal of grassland resources, as well as considering the local economic benefits and ecological effects, LG offers a valid grazing management practice to enhance ecosystem carbon storage. Our research incorporating grazing intensity into shrub ecosystem carbon sinks and sources has great implications for grassland ecosystem carbon management.

## Supplemental Information

10.7717/peerj.12771/supp-1 Supplemental Information 1 Raw data

## Additional Information and Declarations

### Data Availability

The following information was supplied regarding data availability: The raw data are available in the Supplementary File.
